# Round the Hospitals

**Published:** 1917-11-17

**Authors:** 


### ROUND THE HOSPITALS.

The reproduction of the Reredos in King's College Hospital Chapel to the sacred memory of Sister-Matron Katharine Monk is well chosen. This all who refer to Revelation 22, first seven verses, will agree. Few subjects more suitable to hospital service and its incidence can be suggested. The work has been beautifully executed, and the Reredos now placed in the chapel as an altar-piece is one to study and wonder at. The skill with which the New Jerusalem is depicted, the clearness secured in so small a space, and the absence of crowding achieved by the artists and producers, are really wonderful and most suggestive. A description of the Reredos and what it portrays is clearly set out in the brief statement we have printed on either side of the reproduction. We hope every reader will read this and then turn to the Book of Revelation, read the 22nd chapter, and so apprehend the picture and its teaching. If all do this they will be grateful for the wonderful skill and marvellous work which Messrs. J. Powell and Sons, of Whitefriars, have put into this beautiful Reredos. The walls of the chapel chancel are in the condition in which they were left by the builders recently, on the completion of their work. We can assuredly hope that Lord Hambleden and the friends of King's College Hospital will make it their business to complete the decoration of the chancel without avoidable delay, and so contribute to the striking effects for good, and deepen the inspiration, which every visitor to and worshipper in the chapel may receive, through the Reredos and its teachings. The excellent results produced by the frame of the panel in "Verde Antico" marble, with an inner frame of alabaster, are so inviting and complete that, we hope they may form a design which will be adopted in future, wherever possible, for altar-pieces, memorial tablets, and bas-relief reproductions in marble. All who visit King's College Hospital chapel should examine the Reredos, which is lighted by a window in the eastern and western walls, so that at certain hours on a fine day the rising and setting sun illuminates the Reredos with fine effect. Special note should be taken of the inscription at the bottom of the picture, with the cross of St. John on the right and an open unsealed book on the left of the marble bearing the words: "Blessed are they that do His commandments that they may have right to the Tree of Life and may enter in through the gates into the City." It would have gladdened our lamented friend, Katharine Monk, to have seen and studied out for herself the beautiful altar-piece erected to her memory. She was truly a wonderful woman in many respects, and her twenty years' association with King's College Hospital, and the fruits of her work when they come to be described, as we hope they may be, cannot fail to renew the inspiration which her nurses and intimate friends derived from their association with her. The Reredos to her memory should prove a shrine of infinite value to the progress and development of British nursing and nurses for all time. Our American sisters will certainly visit it with reverence to their own uplifting.

---

Everyone is rejoicing at the steady progress of the College of Nursing, and the stride forward which has come to it during the last few weeks. All members of the nursing profession will welcome the following clear declaration of Sir Arthur Stanley on a matter which has attracted much interest and is now happily placed upon a basis which will be welcomed by all parties whom it may concern. Sir Arthur Stanley's declaration is as follows:—"Our endeavour will be to draw a clear line between trained nurses and V.A.D.s, and to encourage such of the latter as are suitable to obtain their three years' certificate from a general hospital, which will enable them to become members of the College of Nursing."

---

In our review of the happenings in connection with the Royal British Nurses' Association last week we pointed out that the views of the College of Nursing on the present position had not yet been published, so there was "still time, all too brief as it is, for reconsideration on the part of the sixteen nurses who have delayed the work of the College and placed the Royal British Nurses' Association and its Royal Charter in what amounts to a *cul de sac.*" We have since received a letter from the College of Nursing, Limited, which will be found on page 142, in which it is pointed out that the Charter of the Royal British Nurses' Association, as it stands, gives to that Corporation powers which are admittedly quite inadequate to the present needs of the nursing profession, and that the Supplemental Charter was therefore a necessity. It is added that the alterations suggested by the Privy Council to the Supplemental Charter and Bye-laws do not, in the judgment of the Council of the College, modify that document as agreed between the College and the Association in any substantial sense. It is further pointed out that the Council of the College of Nursing is anxious the College should be established at the earliest possible date on a representative basis, and it was only because of its desire to meet the demands of the R.B.N.A. that it accepted the proviso, as a condition of the amalgamation, that the Joint Council set out in the Supplemental Charter should hold office for two years. This proviso has already proved a serious impediment to the College in its policy of securing State Registration for Nurses, and the Council prefers to abide by the terms of its own constitution, under which the Council becomes elective in April next. In its early days it was of some importance to the College to seek to ally itself with the Association, and of importance to the Association to obtain enlarged powers, the new infusion of energy, and increased membership which the proposed amalgamation, under the title of the Royal British College

(*Continued on page* 148.)

**ROUND THE HOSPITALS**—(*continued*).

of Nursing, would have brought to both. So far as the College of Nursing is concerned the position to-day is that it is now well known, and that an appeal for funds on its behalf has met with the widest sympathetic reception by the press and public, so that the advantage to the College of amalgamation becomes less obvious. The College is still prepared, however, to take the further steps necessary to give effect to the amalgamation, providing the terms of its agreement with the Association are fulfilled. If amalgamation is not to come about through the action of the R.B.N.A., the Council of the College asks that of the R.B.N.A. to regard the negotiations as practically at an end, and to refrain from incurring further expenditure in regard to it. The Council of the College sincerely regrets that its hopes of union with the R.B.N.A. should have been frustrated, and that it should not have the help it anticipated from the members of that Association, whom it hoped to welcome on the Council, in prosecuting the important tasks which have been delayed in the hopes of a joint agreement and professional unity.

---

IT will be matter indeed for regret, in view of the clear misunderstandings under which, as we demonstrated last week, the sixteen members of the Royal British Nurses' Association whose action has created the present *impasse* appear to have laboured, that the scheme of amalgamation which was entered upon with such high expectations on both sides should have failed to materialise. On the other hand., British nurses will immediately benefit if they have secured to them the College of Nursing, resting at once on a representative basis. This will give them the democratic control and other advantages which the great body of trained British nurses wisely and properly desire to secure.

---

THE British Women's Hospital Committee are making steady progress with the Nation's Fund for Nurses. In exact proportion to the intimate knowledge of the circumstances of the war, the place filled with such courage and efficiency by members of the nursing profession, and all that such service has meant to our wounded and sick warriors, is the conviction that the College of Nursing has come at the right moment, and that it must be promptly made strong enough in every sense to enable the nurses to manage their own affairs on a democratic basis, with the co-operation of all sections of the public, and the technical and administrative aids which their trusted friends can afford them in the organisation and placing of the College upon an all-embracing and enduring basis. General Sir Alfred Keogh, G.C.B., Director-General of the Army Medical Services, has expressed to Sir Arthur Stanley the earnest hope that the College of Nursing will be carried out and made good, and that Sir Arthur will give it his full sympathy and active help. Sir Alfred Keogh adds: "Those of us who know intimately the nature of the services rendered by the members of the nursing profession will wish to encourage in every way whatever scheme may be put forward. As one who has perhaps had the best opportunity of knowing what the profession has accomplished, and what it has enabled the administrative and other branches of the medical profession to accomplish, in the many campaigns now being undertaken, I earnestly hope the greatest success will attend your efforts" on behalf of the College and the Nation's Fund for Nurses.

---

GENERAL THE RIGHT HON. J. C. SMUTS, K.C., M.L.A., Minister of Defence, Union of South Africa, Colonial Secretary, Transvaal, in a letter to Lady Cowdray, treasurer of the Nation's Fund, congratulating her upon the success of the British Women's Hospital Committee in the matter of the Star and Garter Home, expresses the hope that she will be equally successful in her second venture, the Nation's Fund for Nurses, "which is one which deserves cordial support." We do not believe there is a single household in the land which will not desire spontaneously to give something at once to the Nation's Fund for Nurses. Christmas Day is a very sacred day in war-time to British households and families, and Christmas 1917 should move both mother and father and the olive branches to make a memorable feature of their Christmas dinner, by each adding a gift to the household contribution to the Nation's Fund for Nurses, and dispatching it by the same night's post to Lady Cowdray, the treasurer, at 16 Carlton House Terrace, London, S.W. 1. General Smuts has already, or may, transmit this idea to South Africa, where family life is a very real and inspiriting influence. It may be taken for granted that the College of Nursing and the Nation's Fund, in close association together, will be welcomed, appreciated, and whole-heartedly supported throughout the British Dominions beyond the seas.

---

OUR anticipation that the press of all shades of opinion would extend generous support to the British Women's Hospital Committee's movement to raise money for the College buildings, its scholarships, and educational work for nurses has been quickly fulfilled. Week by week important newspapers are making the Nation's Fund for Nurses known and appreciated. We have now to include in the list of the nurses' friends in the British press, which appeared in our issue of November 3, the *Morning Post*, the *Daily Mail*, the *Morning Advertiser*, the *Newcastle Journal*, the *Guardian*, the *Christian Commonwealth*, the *Schoolmistress*, *Country Life*; the *Ladies' Field*, the *Ladies' Pictorial*, the *Queen*. the *Sketch*, the *Tatler*, and the *Weekly Irish Times*. We shall welcome an intimation, or a copy of the paper containing matter in support of the Nation's Fund for Nurses, if the editors will kindly forward it, so as to enable us to keep the nurses informed of their friends who are working for them in the British and Empire press. The British press was never more powerful than it is to-day, and its power was never better employed or devoted to an objective which has commended itself more directly and completely

**ROUND THE HOSPITALS—**(*continued*).

to the sympathies and welcome of all classes throughout the country than has the British Women's Hospital Committee's work for British nurses and British nursing. The tare sowers have done their best to injure this movement for nurses, but the editors have, as we anticipated, found the w.p.b. a most useful and handy ally in effectually disposing of matter which, on the face of it, was prejudiced and opposed to the will and interests of the people of these islands.

The Angel matinée at the Palace Theatre, organised by the British Women's Hospital Committee, proved a gratifying success. Thanks to the liberality of Lady Cowdray, who defrayed all the expenses, a sum of £1,070 has been added to the Nation's Fund for Nurses.

We are glad to state, on the authority of Lady Robertson, that Her Majesty the Queen has given one more instance of her untiring activity and interest in furthering everything which can minister to the comfort and uplifting of our warriors. At the Queen's suggestion a scheme is now being evolved to help completely and practically the disabled and discharged soldiers—and sailors, too, we hope. Such a scheme exhaustively conceived and perfectly organised will be one more bond in uniting all classes of the community in the most cordial and whole-hearted co-operation for and with those who, without any thought of their own glorification or reward, have offered their lives and sacrificed much that makes life at its best, in defence of the nation and the maintenance of the Empire.

Once again the season has come round for the Women's Associations, that throughout the country do such invaluable work for British hospitals, to give an account of their stewardship. Lady Bryce on the 7th instant presided at the seventeenth annual general meeting of the University College Hospital Ladies' Association in the College Library, when Lady Elizabeth Dawson reported it now had a total of 821 members. During the year 1,146 garments had been contributed for the use of patients, and the Association had been able to pay over to the hospital £881, the total receipts, according to the statement of Lady Godlee, the treasurer, amounting to £1,507. Sir Ernest Hatch informed the ladies that the hospital's expenditure amounted to £45,000, and 55,000 out-patients had been treated.

In Ireland, where the movement has spread, Linen Guilds are popular. At a meeting of the Royal City of Dublin Hospital, the Primate in the chair, which was largely attended by ladies, Miss Eddison, who is the matron of the hospital and honorary secretary of the Guild, made an interesting report. She stated the Guild had been inaugurated by the Countess of Pembroke in 1910, and had practically since that time supplied the hospital with quilts, sheets, pillow-cases, blankets, towels, shirts, nightgowns, bed-jackets, frocks, hot-water bottle covers, operation stockings, and so forth. Unfortunately the Guild was not in funds at the moment, having spent £141 on linen this year and received so far only £94. The Guild was urgently in need of an immediate stock of linen, which would last for the next two years, and the money available, £50, was wholly inadequate to effect this object. The number of sheets in daily use, and in circulation in the hospital, was 1,037. One of the first lessons a nurse had to learn was to keep her patient clean and therefore comfortable, which could not be done without a generous supply of bed-linen, which had become an urgent necessity at the moment. The Primate said, "Man thinks that he knows, but woman knows better." The Guild afforded proof of what could be done by the organised effort of women labouring regularly and patiently. The hospital at the meeting of whose Linen Guild he was presiding was a clinical school, where nurses and doctors learnt lessons the benefits of which the people reaped in their own homes. Love was a chain that linked Heaven with the things of the earth, but there was a gap in the chain, which was filled by the human hand, and the Linen Guild helped to fashion that hand by the principle of love that governs it. The men had an opportunity to supply the £100 urgently needed to enable the Guild to meet the pressing necessities of the Hospital Committee at the moment for linen and other things. It is not thinkable that the men of Dublin will not step into the breach for an Irish hospital, as the men practically everywhere else throughout the Empire have done and are doing regularly as a privilege which they would not miss. The meeting congratulated Miss Eddison on the distinction she had received as the recipient of the Royal Red Cross.

Several of the large cities in Scotland are centres where the Nursing Home has fulfilled many useful purposes, and are largely availed of by all classes of people in the day of serious illness, especially those requiring surgical aid. One of the most successful and well-known Homes is that known as the M'Alpin Nursing Home at Glasgow, which was founded by Miss Agnes M'Alpin in 1874, the present building being opened in 1908. Miss M'Alpin, who is described as "the pioneer of homes for training nurses," has just died at the age of ninety-two. She had associated with her her sister, Miss Jessie M'Alpin, who still survives, and they fought a brave fight and persevered until the enterprise was crowned with professional and lay approval.

Nursing Homes in their day have fulfilled their purpose, and we look forward to the time now approaching when those which continue to exist will be registered, and regularly inspected by the officers of the County or Municipal Authorities. The time should not be far distant when they will be superseded by pay hospital accommodation in connection with the chief hospitals, and so help

**ROUND THE HOSPITALS—**(*continued*).

to complete the medical organisation on the best lines, for the reception and treatment of all sick persons, from the highest to the humblest, in accordance with their means and requirements.

IN our issue of December 23, 1916, page 252, we gave some account of the high value and importance, in an educational sense, of the secondary school system for girls in the United States. This system was organised to fit them, by training, and the inculcation of a high standard of personal effort, to prepare themselves for the life and work which awaits them when their school careers are ended. The thoroughness of the American system, and its splendid organisation, so far as children are concerned, receives a further confirmation in a paper by G. Ivy Sanders, who records that on August 29 of this year the War Council at Washington approved a plan for junior Red Cross membership, whereby practically every private or public school became a school auxiliary of the American Red Cross, and every child attending that school automatically became a member of the junior Red Cross, entitled to wear the white button of membership with the Red Cross emblazoned on it. Under this scheme each school has a fund raised by contributions from interested people, earned individually, or in groups or classes, and by entertainments given by the children, which supplies the money to purchase wool, flannel, dress, and other materials to enable the children to be organised for work. The Commission of Education has ordered the regular sewing classes in the schools to substitute for the old plan of making useless or purely ornamental things, work for the Red Cross. The school authorities retain direct and entire control of the schools and Red Cross junior membership, but co-operate in general matters with the local Red Cross branch. The Principal of each school becomes Chairman of the School Auxiliary and appoints the Treasurer of the local School Supply Fund.

THE thoroughness of the system followed is indicated by the fact that the organisation includes committees for membership, school activities, distribution of materials, assembling of materials, pattern and inspection, packing and shipping. In New York State alone nearly 2,000,000 children are already employed in making articles for the desolated children of the Allied countries—hospital supplies, bed-linen, pyjamas, socks, surgical dressings, bandages, knitted jackets, mufflers, and other articles required by the Red Cross. The boys, too, are making crutches, splints, footstools, leg-rests, and countless other things included in hospital equipment. Already 145 American cities have begun work for the Red Cross, and its value can be estimated from the fact that there are more than 22,000,000 school children in the United States. It will be interesting to know what steps, if any, the Boards of Education in Great Britain have taken in this direction, or whether we are to wait for the new Education Bill to become law before our children will be organised on some complete plan.

☞ IT is our invariable practice to pay for all items of news which we may publish. The minimum payment is 5s. Paragraphs of about twenty lines in length—that is to say, of 150 words or less—are preferred. Contributions should be addressed to The Editor, THE HOSPITAL, 28 and 29 Southampton Street, Strand, London, W.C. 2., and, to be in time for the current week's issue, should reach him by the first post on Mondays.

**For Nursing Affairs in Ireland, see p. 144; Matrons' and Sisters' Appointments, see p. 152.**

